# Daith Piercing: Wonder Treatment or Untested Fad?

**DOI:** 10.7759/cureus.6978

**Published:** 2020-02-13

**Authors:** Priyanka Bhandari, Eukesh Ranjit, Amit Sapra, Dean Davis, Careyana Brenham

**Affiliations:** 1 Family Medicine, Southern Illinois University School of Medicine, Springfield, USA

**Keywords:** chronic migraine, body piercings, daith piercing, headache, resistant migraine, complications of piercing, alternative medicine, headache treatment

## Abstract

Daith piercing is a form of body piercing that involves the crux of the ear’s helix. While daith piercing has been used as an esthetic piercing since the 1990s, it is gaining popularity in the general population as an alternative treatment in chronic headaches, especially migraines. Despite its use, the evidence is currently lacking. Postulated hypotheses include vagal neuromodulation vs. placebo effect. We present a case of a 47-year-old female patient suffering from refractory cluster headache who underwent daith piercing. We aim to raise awareness among the general practitioners of this health-related practice prevalent in the community.

## Introduction

Daith piercing is a form of ear piercing located at the crux of the helix of the ear. While the esthetic piercing of the crux of the helix of the ear has been seen in various cultures around the world, the term "daith piercing" (from Hebrew: דעת da’at: knowledge) originated in alternate lifestyle communities in the 1990s [1]. It was shrouded in a belief that the piercing acts as a gateway for only knowledge to pass through the ear [2]. The piercing gained mainstream popularity in the following years and has been subsequently used for migraine as an alternate treatment by some patients with anecdotal patient reports online suggesting an improvement in symptoms [3]. This case illustrates a current common health-related practice in the community, the awareness of which could help the provider to manage this condition better. 

## Case presentation

Our patient is a 47-year-old right-handed female who presented to the Family Medicine clinic for evaluation of her headaches. The patient stated that she started having headaches around the age of 10, which became frequent around her teens and early 20s. She typically described her headaches as a pressure sensation that would last from 10 to 30 minutes and occur about 6-8 times per day. The pain was mostly in the occipital or the periorbital area. She denied any tearing but did endorse nasal congestion on the side of the pain. Associated symptoms included nausea, photophobia, and phonophobia. She denied visual aura, ptosis, diplopia, blurry vision, nausea, vomiting or sensitivity to smell, dysphagia, dysarthria, dizziness, weakness, numbness, or tingling or any other neurological deficit. She could not identify any triggers but stated that initially, the headaches would only occur during the day. Later, the headaches also started occurring at night and waking her up from sleep. She denies a family history of headaches. She usually drinks two cups of coffee per day, and this practice has not changed in many years. There was no preceding history of any injuries, falls, or head trauma. 

She saw a neurologist in her teens and was diagnosed with cluster headaches. The headaches improved in her late 20s and 30s but recently have worsened again. She states that about four years ago, she had headaches in the left occipital region, which lasted almost six weeks. She tried multiple over-the-counter medications such as Excedrin without any relief. Her prior primary care provider had also tried topiramate and amitriptyline without significant benefit.

**Figure 1:**
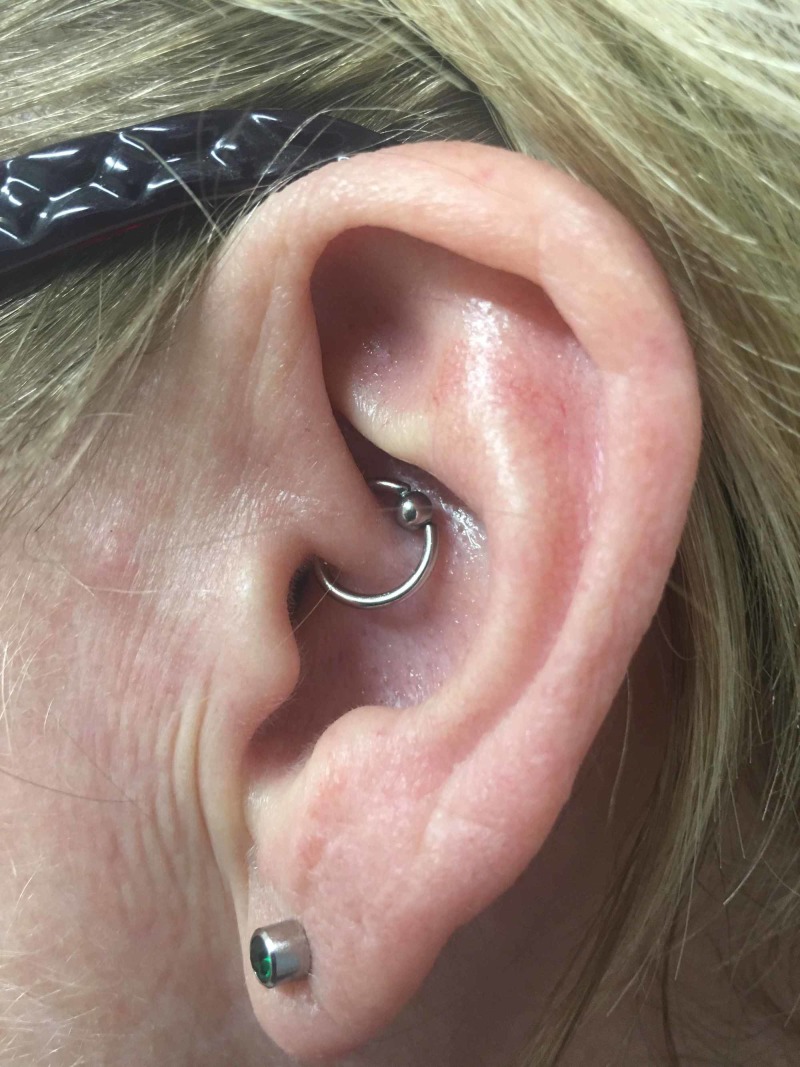
Daith piercing located at the crus of the helix in the left ear

Later, she could not follow up with her neurologist or headache specialist due to insurance issues. She then decided on getting daith piercing on the left side after hearing about it from her colleagues and friends. She underwent the piercing at a local tattoo shop in her town and stated that the left-sided headaches completely resolved almost immediately (Figure 1).

**Figure 2:**
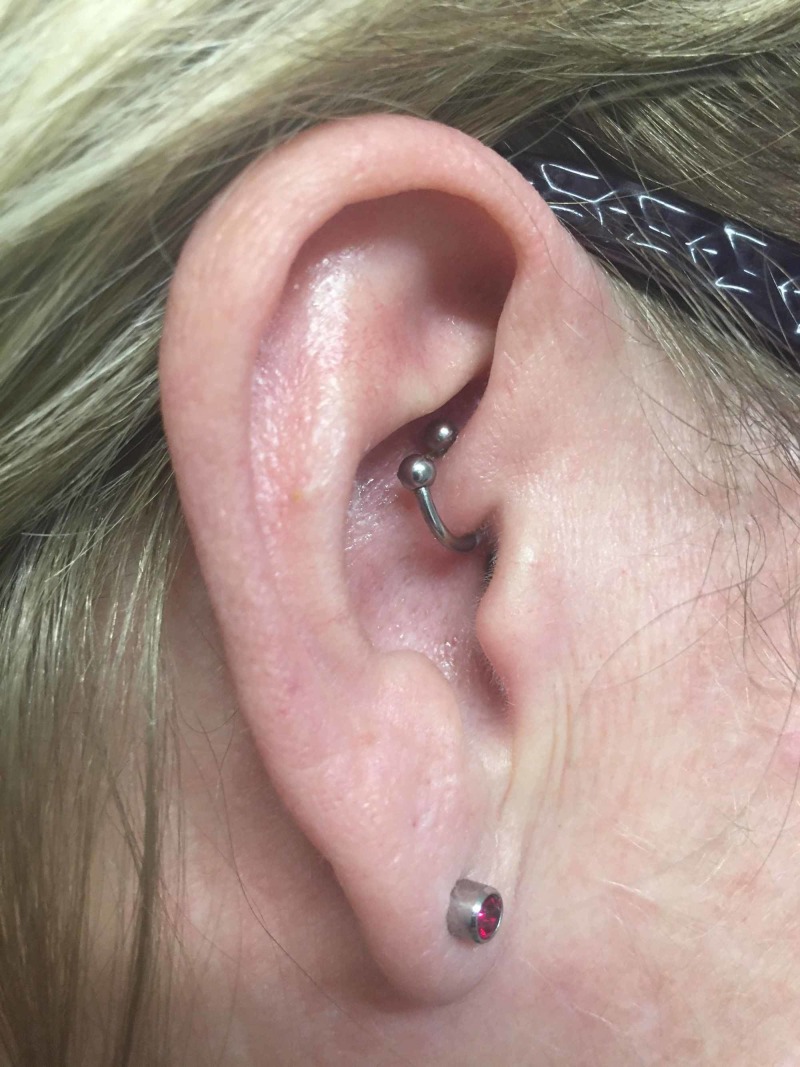
Daith piercing located at the crus of the helix in the right ear

She was asymptomatic till the beginning of March 2019 when the headaches returned but now on the right side. It was once again in the right occipital region. She stated that she would have episodes of headaches that lasted for almost four weeks and affected her activities of daily living. She had a daith piercing done on the right side, and the headaches resolved (Figure 2).

The headaches gradually came back on the right side in December 2019 and lasted for almost two and a half weeks, after which she came to see us in the clinic. She stated that in the last two and a half weeks, she had tried multiple over-the-counter pain medications without much relief. She had been missing work being incapacitated with pain. She denied having used beta-blockers like propranolol or metoprolol, GABAergic agents like gabapentin, pregabalin, or anticonvulsants like lamotrigine or divalproex.

On examination, she was normocephalic. There were no palpable masses or tenderness on the face or head. Her neck exam was unremarkable, with no tenderness, and her neck movements were full. There were no meningeal signs. Spurling’s sign was negative. The rest of the neurological exam was within normal limits. Her visual acuity was reported 20/20 in each eye. The patient was also advised to see her optometrist immediately for a detailed examination, and there was no evidence of papilledema. We started the patient on verapamil 40 mg twice a day, as well as rizatriptan 10 mg PRN (with a max of three tabs in 24 hours) and ordered a computed tomography (CT) of her head without contrast. A referral to a neurologist was also made.

On the subsequent appointment, she reported a decrease in both the intensity and frequency of her headaches. We increased the dose of her verapamil to 40 mg every eight hours. She subsequently saw the neurologist who gave her a differential diagnosis of cluster headaches versus hemicrania continua. She was advised to continue with her current dose of verapamil.

**Figure 3:**
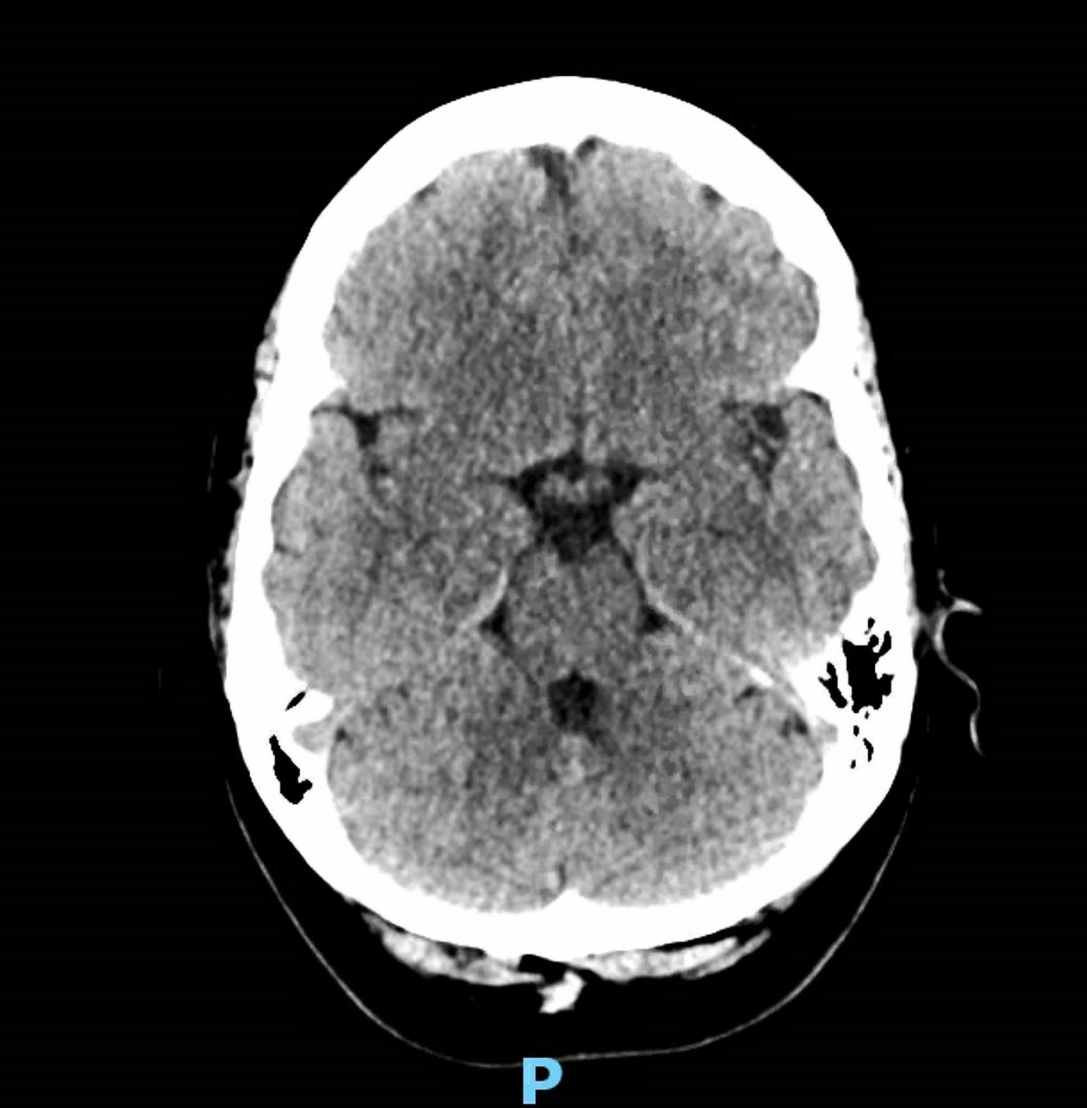
Non-contrast computed tomography head revealing no acute intracranial findings

Non-contrast CT head revealed no acute intracranial findings (Figure 3).

Our patient continues to take some of her medications. However, she reports a marked improvement in her symptoms after daith piercing and is motivated to cut down on her medications.

## Discussion

Incidence/prevalence

The scientific literature on the subject is limited. Daith piercing is being conducted in non-medical facilities on a routine basis. Also, there is no data on the number of daith piercing done primarily for medical reasons. Age and gender-related data could not be found specifically for daith piercing due to a paucity of literature. A survey among graduate students in the United States found 42% of men and 60% of women surveyed had body piercings [4].

A Pubmed search of “daith piercing” and “daith” revealed only one case report. Google Scholar search revealed 35 results for “daith piercing.” After omitting duplicates, the mere mention of term daith piercing and non-medical entries, four entries (one poster, one case report, one symposium presentation, and one research abstract) were found. Besides these four, a survey in England, "International surveys of the effects of Daith piercing on migraine," conducted by the London Migraine Clinic found high satisfaction among people with migraine who underwent daith piercing; 75% of the patients who participated in the survey said their migraines were “greatly improved" [5].

Epidemiology

Body piercing practices have their origins in various ancient cultures, including the Indian, Egyptian, Chinese, and Roman cultures. Recent years have seen a dramatic rise in multiple body piercing in Western cultures, including the United States [6].

Mechanism of action

Even though no clear mechanism of action is known, a multifactorial effect has been proposed [7]. Vagal neuromodulation has been hypothesized to modulate pain pathways of headache [8]. Vagal stimulation can exert its effect through multiple pathways, including inhibitory action on nociceptive neurons in the caudal trigeminal nucleus, modification of cortical excitability through various connections in the pain matrix and activation of the descending inhibitory pathways [9]. Non-invasive stimulation methods of vagus via electrode placement on the ear, as well as acupuncture therapies, support the possible effectiveness of this therapy. It has been hypothesized that the sensory stimulus provided by piercing the site modulates the nociceptive input via the trigeminovascular system, and hence alters the pathway [7]. Possible placebo effect has also been proposed by headache specialists [10].

Complications

According to a study, up to 35% of ear piercings develop complications [11]. Like any other ear piercings, potential complications of daith piercing include, and can broadly be classified into infective and non-infective complications. 

Infectious complications ranging from perichondritis, cellulitis, abscess to infective endocarditis have been reported for ear piercings [11,12]. Bloodborne infections like human immunodeficiency virus (HIV), hepatitis B, hepatitis C, and tetanus are possible. There is no specific guideline for antibiotic prophylaxis for patients undergoing body piercing. 

Non-infective complications include immediate pain, swelling, laceration, erythema, as well as allergic reaction, contact dermatitis, deformity, hypertrophic scar, keloid, and perichondritis [11]. Because the piercing involves a part of cartilage with poor blood supply, delayed healing could be seen. 

One case report presents a failure of daith piercing in a patient with chronic migraine [13].

Recommendations

Due to limited data availability, there are no standard recommendations supporting the use of daith piercing [14]. The American Migraine Foundation does not recommend its use considering risks of infection and pain. The foundation also suggests the possibility of a placebo effect [15]. A German guideline for the treatment of migraine recommends against the use of secondary due to lack of evidence [16].

## Conclusions

Even though no clear mechanism of action of daith piercing is known, a multifactorial effect has been proposed. While daith piercing is seen as an inexpensive, simple non-clinical procedure, potential side effects persist. Possible infectious and non-infectious complications should be explained to patients contemplating the procedure. A lack of standard recommendations should be disclosed. Very scant medical literature exists on the subject at the moment, and more research and scientific studies are warranted to see its long term effect. 
